# Potential mechanisms by which microbiota-accessible carbohydrates regulate hepatic lipid metabolism in MAFLD via the gut-liver axis

**DOI:** 10.3389/fmicb.2026.1898227

**Published:** 2026-08-18

**Authors:** Jingyi Peng, Xiaojiao Gao, Guanglei Chen, Ankangxin Yan, Qi Yu, Weiyi Tian, Kun Cai

**Affiliations:** 1 Guizhou University of Traditional Chinese Medicine, Guiyang, Guizhou, China; 2 Guizhou Nursing Vocational College, Qiannan Buyi and Miao Autonomous Prefecture, Guizhou, China; 3 Guiyang Kangyang University, Guiyang, Guizhou, China

**Keywords:** 5-HT, *Akkermansia muciniphila*, gut-derived multi-level metabolic signaling network, gut-liver axis, metabolic dysfunction-associated fatty liver disease, microbiota-accessible carbohydrates

## Abstract

Metabolic dysfunction-associated fatty liver disease (MAFLD) is a prevalent chronic metabolic liver disorder, whose onset and progression are closely associated with dysregulated lipid metabolism, chronic inflammation, and intestinal microbial dysbiosis. The gut and liver are anatomically and functionally connected via the portal vein system, through which gut-derived metabolites, inflammatory mediators, and neuroendocrine signals are preferentially delivered to the liver, thereby influencing hepatocellular lipid metabolism and hepatic sinusoidal immune responses. Dietary complex carbohydrates, particularly Microbiota-accessible carbohydrates (MACs), are fermented by gut microbiota in the colon. By reshaping luminal carbon source distribution, microbial composition, and metabolic output, MACs modulate gut-liver axis function. This review summarizes the potential mechanisms by which MACs influence MAFLD via the gut-liver axis. Structurally complex and slowly fermentable MACs may prolong carbohydrate availability in the distal colon and reduce microbial shifts toward proteolytic fermentation, thereby decreasing the production of potentially harmful metabolites such as ammonia, phenols, p-cresol, and hydrogen sulfide. Short-chain fatty acids (SCFAs) produced from MAC fermentation, particularly acetate and propionate, as well as microbiota-modified bile acids, can enter the portal circulation and regulate hepatic metabolic signaling, thereby participating in the control of *de novo* lipogenesis, fatty acid oxidation, and inflammatory responses. In addition, MACs may improve intestinal barrier integrity and modulate 5-hydroxytryptamine (5-HT, serotonin) metabolism through effects on key microbial taxa such as *Akkermansia muciniphila* and regulation of SERT-dependent pathways. Gut-derived 5-HT may further participate in hepatic lipid metabolic regulation via pathways such as HTR2A-PPARγ signaling, although this mechanism remains to be fully validated. Overall, MACs may influence MAFLD progression through coordinated regulation of gut microbiota composition, gut-derived metabolites, and intestinal barrier function. However, their clinical translation still requires further validation through human studies and comprehensive safety evaluation.

## 1 Introduction

Metabolic dysfunction-associated fatty liver disease (MAFLD; formerly termed non-alcoholic fatty liver disease, NAFLD) is one of the most prevalent chronic liver diseases worldwide (Riazi et al., 2022). Its global prevalence approaches 30%, and the age of onset has shown a concerning downward trend in recent years (Eslam et al., 2020a,b; Powell et al., 2021). MAFLD is characterized by hepatic steatosis and may progress to hepatic inflammation and fibrosis. Moreover, it frequently coexists with type 2 diabetes mellitus, obesity, and cardiovascular disease, thereby imposing a substantial public health burden (Chan et al., 2022; Lazarus et al., 2022). Current evidence suggests that the pathogenesis of MAFLD involves complex interactions among multiple pathological processes, including insulin resistance, oxidative stress, endoplasmic reticulum stress, and chronic low-grade inflammation (Buzzetti et al., 2016; Targher et al., 2021). However, effective pharmacological therapies capable of consistently reversing disease progression remain limited, and long-term adherence to lifestyle interventions is often suboptimal (Francque et al., 2021). Consequently, identifying upstream and modifiable pathogenic factors represents a promising strategy for disease prevention and management. In this context, gut microbial dysbiosis has increasingly been recognized as an important therapeutic target in the development and progression of MAFLD (Liao et al., 2026).

The gut microbial ecosystem plays a pivotal role in metabolic diseases through its regulation of nutrient absorption, energy homeostasis, and endocrine function in the host (Sonnenburg and Bäckhed, 2016). The intestine and liver are closely interconnected both anatomically and functionally, collectively forming the gut-liver axis (Tripathi et al., 2018). Approximately 70% of the hepatic blood supply is derived from the portal vein (Albillos et al., 2020). Consequently, nutrients absorbed from the intestine, microbiota-derived metabolites such as short-chain fatty acids (SCFAs) and secondary bile acids, as well as gut-derived antigens, are preferentially delivered to the liver via the portal circulation (Zhu et al., 2024). Under conditions of gut microbial dysbiosis, the production of harmful metabolites and pathogen-associated molecular patterns (PAMPs) may increase, allowing these factors to reach the liver through the portal vein and contribute to local hepatic inflammation and metabolic dysfunction (Tilg et al., 2016; Qin et al., 2025). Therefore, disruption of gut-liver axis homeostasis is increasingly regarded as a key mechanism driving MAFLD progression (Singh et al., 2026).

Both clinical and experimental studies have demonstrated that MAFLD-associated alterations in the gut microbiota are commonly characterized by reduced microbial diversity and decreased abundance of specific metabolically beneficial taxa (Loomba et al., 2017). For example, a reduced abundance of *Akkermansia muciniphila* has been associated with more severe hepatic steatosis and inflammatory injury in several studies (Depommier et al., 2019; Xu et al., 2020). Gut microbial dysbiosis may influence hepatic function through at least two major pathways. First, impairment of the mucus layer and disruption of intestinal tight junctions can increase intestinal permeability, thereby facilitating the translocation of bacterial-derived molecules such as lipopolysaccharide (LPS) and promoting metabolic endotoxemia (Cani et al., 2007). Second, dysbiosis may disturb gut neuroendocrine homeostasis, particularly through alterations in the expression and function of the serotonin transporter (SERT) (Yano et al., 2015). Reduced SERT activity may decrease intestinal 5-HT reuptake, resulting in elevated levels of free serotonin (5-hydroxytryptamine, 5-HT). This gut-derived 5-HT may subsequently enter the liver through the portal circulation and contribute to dysregulated hepatic lipid metabolism (Cao et al., 2017; Choi et al., 2018).

Given the critical involvement of gut-derived signals in MAFLD pathogenesis, modulation of host–microbiota interactions has emerged as a promising nutritional intervention strategy. Microbiota-accessible carbohydrates (MACs) represent a class of complex dietary carbohydrates that cannot be completely degraded by host digestive enzymes and therefore reach the colon, where they serve as substrates for microbial fermentation (Deehan et al., 2017). Owing to differences in their physicochemical structures, distinct MACs exhibit varying degradation rates and fermentation sites within the colon, thereby influencing carbon source distribution, microbial composition, and microbial metabolic function, ultimately reshaping the metabolite profile of the gut ecosystem (Yang et al., 2023; Wu et al., 2024). On the one hand, MACs may promote the growth of beneficial microorganisms such as *A. muciniphila*, improve intestinal barrier integrity, and potentially influence SERT-mediated 5-HT reuptake (Bhattarai et al., 2018; Depommier et al., 2019). On the other hand, microbial fermentation of MACs can enhance SCFA production, modulate bile acid metabolism, and reduce the generation of potentially harmful metabolites, including ammonia and endogenous ethanol (Li et al., 2021). Collectively, these changes may alter the profile of gut-derived metabolites entering the portal circulation, thereby influencing hepatic lipid metabolism, inflammatory responses, and the progression of MAFLD.

**FIGURE 1:**
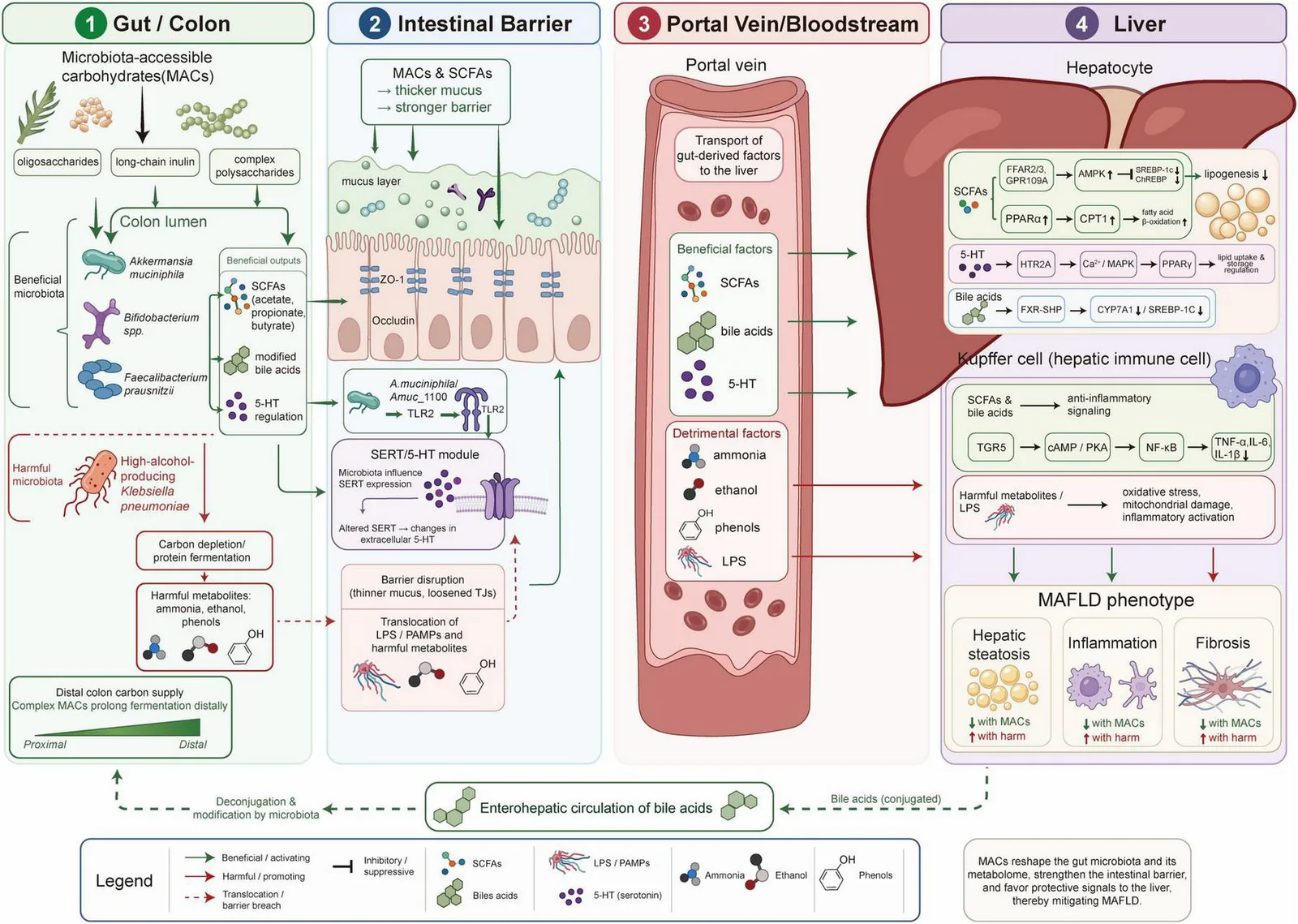
Remodeling of the gut-liver axis by Microbiota-accessible carbohydrates (MACs) in metabolic dysfunction-associated fatty liver disease (MAFLD).

In this review, we summarize the potential mechanisms through which MACs influence MAFLD via the gut-liver axis from the perspectives of dietary complex carbohydrates, gut microbiota, and hepatic lipid metabolism. We further discuss how SCFAs, bile acids, and selected potentially harmful microbial metabolites traverse the intestinal mucosal barrier and subsequently affect hepatic metabolic and immune signaling through the portal circulation. Finally, we examine the potential regulatory effects of these gut-derived signals on hepatocellular lipid synthesis, fatty acid oxidation, and Kupffer cell-mediated inflammatory responses. An overview of the potential mechanisms linking MACs, the gut microbiota, the intestinal barrier, and hepatic metabolism is presented in Figure 1. By integrating current evidence, this review aims to provide a theoretical framework for the development of microbiota-targeted nutritional interventions for MAFLD.

## 2 Structural characteristics of dietary complex carbohydrates and their fermentation distribution in the colon

Dietary complex carbohydrates, namely MACs, cannot be fully degraded by host digestive enzymes and therefore reach the colon, where they serve as important fermentation substrates for the gut microbiota (Chen S. et al., 2022; Wu et al., 2024; Zhou et al., 2025b). The utilization of MACs in the colon does not occur uniformly; instead, it is jointly influenced by their molecular structure, degradability, and the enzymatic repertoire of the microbiota. Different types of MACs exhibit distinct degradation rates, fermentation sites, and metabolite profiles within the colon, which further shape the spectrum of gut-derived metabolites entering the liver via the portal vein (Zhu et al., 2022; Ni et al., 2023).

### 2.1 Molecular structure determines the degradation rate and fermentation site of MACs

The gut microbiota encodes a diverse array of carbohydrate-active enzymes (CAZymes), enabling the degradation of a wide range of host-indigestible complex carbohydrates. However, the extent to which substrates can be efficiently utilized largely depends on their molecular structures. The degree of polymerization (DP) of polysaccharide backbones, monosaccharide composition, glycosidic bond types, branching degree, and spatial conformation all influence microbial recognition, binding, and degradation efficiency (Yang et al., 2013; Wang et al., 2019; Bai et al., 2020; Vinelli et al., 2022).

In general, oligosaccharides with lower molecular weight, lower branching degree, and higher solubility are more readily and rapidly utilized by the microbiota in the proximal colon. In contrast, high-DP or structurally complex polysaccharides, such as long-chain inulin and certain structurally defined pectins, typically require microbial communities equipped with specific polysaccharide utilization systems and coordinated enzymatic activities for degradation (Bao et al., 2020; Yang et al., 2023; Sun et al., 2024; Amadieu et al., 2025). Some structurally complex and slowly degradable substrates are not rapidly consumed in the proximal colon and therefore may extend fermentation activity toward the distal colon. Accordingly, the structural features of MACs not only determine fermentation efficiency but also influence their spatial distribution of utilization and the resulting metabolite profiles along different colonic regions.

### 2.2 Shifts in colonic carbon source availability and generation of harmful metabolites

As luminal contents transit from the proximal to the distal colon, the availability of utilizable carbohydrates generally decreases. The proximal colon typically contains abundant readily fermentable carbohydrates, where microbial saccharolytic metabolism is highly active and produces substantial amounts of SCFAs. SCFAs can lower luminal pH, inhibit colonization by certain pathogens, and act as key gut-derived metabolites entering the portal circulation.

When contents reach the distal colon, limited availability of fermentable carbohydrates may increase microbial reliance on protein and amino acid fermentation (Janssen et al., 2017; Singh et al., 2019; Ji et al., 2020). Accompanied by enhanced proteolytic fermentation induced by MACs deficiency, the production of potentially harmful metabolites such as ammonia, phenol, p-cresol, indole, hydrogen sulfide, and others is increased in the colon. Meanwhile, gut microbial dysbiosis may also promote endogenous ethanol production, collectively disrupting intestinal barrier integrity and exacerbating MAFLD progression through the gut-liver axis. These molecules can be absorbed across the intestinal epithelium, increasing portal venous exposure and thereby potentially affecting hepatic metabolism and inflammatory responses. For instance, excessive ammonia may increase the metabolic burden of the hepatic urea cycle and is associated with enhanced oxidative stress. Certain high-ethanol-producing bacterial strains, such as high-alcohol-producing *Klebsiella pneumoniae* (HiAlc Kpn), can generate substantial endogenous ethanol, which is associated with hepatic lipid droplet accumulation and oxidative stress (Yuan et al., 2019; Lee et al., 2020; Li et al., 2021; Gan et al., 2023; Fan et al., 2025; Ren et al., 2026). Phenolic derivatives such as p-cresol may also impair mitochondrial function and participate in the regulation of Kupffer cell–mediated inflammatory responses (Chen et al., 2021; Tan et al., 2023; Hu M. et al., 2025; Lu et al., 2026; Zhang et al., 2026).

Therefore, carbon source depletion in the distal colon may shift microbial metabolism from saccharolytic fermentation toward proteolytic fermentation, thereby increasing the production of harmful gut-derived metabolites. This process may represent one mechanistic link through which gut microbial dysbiosis contributes to MAFLD progression.

### 2.3 Delayed fermentation and carbon source supplementation in the distal colon

Structurally complex and slowly degradable MACs may reduce rapid carbohydrate consumption in the proximal colon, allowing a portion of carbon sources to be delivered to the distal colon. This shift in fermentation location helps alleviate carbohydrate scarcity in the distal colon and may reduce microbial dependence on protein and amino acid fermentation substrates (Van-Wehle and Vital, 2024).

With sustained carbohydrate availability in the distal colon, acetate- and butyrate-producing bacteria and their cross-feeding relationships may be better maintained. This supports intestinal mucosal barrier function and promotes the expression of tight junction proteins such as Occludin and ZO-1, thereby reducing the risk of bacterial-derived molecules such as LPS translocating through the portal circulation (Jackson et al., 2024; Xie et al., 2024). Meanwhile, adequate carbohydrate supply may reduce the relative intensity of proteolytic fermentation, thereby decreasing the generation of harmful metabolites such as ammonia and phenolic compounds; however, its effects on endogenous ethanol production may depend on specific bacterial strains and dietary contexts (Ornelas et al., 2023; Hays et al., 2024; van Deuren et al., 2025).

Accordingly, the regulation of fermentation sites and carbon source distribution by MACs may influence the gut-liver axis through two mechanisms: (i) supporting intestinal barrier integrity and reducing endotoxin translocation, and (ii) decreasing portal exposure to harmful metabolites, thereby alleviating hepatic metabolic stress and inflammatory activation.

### 2.4 Regulatory effects of complex carbohydrates on key microbial taxa

Clinical observations and animal studies suggest that the relative abundance of *A. muciniphila* may be reduced in MAFLD, and in some studies, this reduction is associated with aggravated hepatic steatosis and inflammation (Gowd et al., 2019; Meiners et al., 2025; Huang et al., 2026). The structural characteristics of different MACs and their corresponding colonic fermentation profiles are summarized in Table 1.

**TABLE 1:** Structural characteristics of different Microbiota-accessible carbohydrate (MAC) types and their colonic fermentation profiles.

MAC type	Molecular structural features	Proximal colonic fermentation	Distal colonic fermentation	SCFA production	Effects on harmful metabolites	Potential impact on the gut–liver axis	References
Oligosaccharides (low DP, low branching, high solubility)	Easily fermentable, low-degree polymerization	Rapid consumption	Limited	Rapid production of acetate and propionate	Limited impact on distal proteolytic fermentation	Rapid delivery of hepatic metabolic substrates, but limited distal colonic protection	Makki et al., 2018; Bai et al., 2020
Long-chain inulin/low-esterified pectin	Medium-to-high DP, moderate branching	Moderate consumption	Extended to distal colon	Increased butyrate with moderate acetate/propionate	Reduced production of ammonia, phenolic compounds, and endogenous ethanol	Sustained carbon source supply, improved mucosal barrier integrity, reduced endotoxin translocation	Bao et al., 2020; Arifuzzaman et al., 2022
Highly esterified pectin/complex polysaccharides	High DP, highly branched, complex spatial conformation	Slow fermentation	Sustained distal colonic fermentation	Increased butyrate with balanced SCFA profile	Effective suppression of proteolytic fermentation	Prolonged distal fermentation, maintenance of cross-feeding networks and key microbial activity	Beukema et al., 2020; Tang and de Vos, 2025

Previous studies have demonstrated that the outer membrane protein Amuc_1100 of *A. muciniphila* can interact with Toll-like receptor 2 (TLR2) on intestinal epithelial cells and participate in the regulation of intestinal mucosal barrier function (Wang et al., 2021; Chen X. et al., 2022; Cheng et al., 2022; Wang et al., 2026). Under certain experimental conditions, *A. muciniphila* may also modulate the local intestinal immune microenvironment and be associated with changes in SERT expression; such alterations may further influence 5-HT reuptake and local homeostasis in the gut (Huang et al., 2023; Yang et al., 2023; Li K. et al., 2024). The key microbial taxa, regulatory effects of MACs, and their potential downstream consequences are summarized in Table 2.

**TABLE 2:** Key gut microbial taxa, regulatory effects of Microbiota-accessible carbohydrates (MACs), and downstream functional outcomes.

Microbial taxa	Functional role	Regulation by MACs	Downstream molecules/pathways	Effects on the gut–liver axis	Clinical relevance	References
*Akkermansia muciniphila*	Mucosal barrier maintenance; modulation of 5-HT signaling	Increased abundance	Amuc_1100 → TLR2 → SERT / 5-HT signaling axis	Improved intestinal barrier integrity and regulation of gut-derived 5-HT levels	Reduced abundance in MAFLD associated with hepatic steatosis and disease severity	Cani et al., 2022
*Faecalibacterium prausnitzii* / butyrate-producing bacteria	Energy source for colonocytes; anti-inflammatory effects	Increased substrate availability for fermentation	GPR109A activation → HDAC inhibition → NF-κB modulation	Increased butyrate production; maintenance of tight junction protein expression	Associated with reduced hepatic inflammatory burden	Miquel et al., 2013
Acetate-producing bacteria (e.g., *Bifidobacterium* spp.)	Provide metabolic substrates for host hepatic metabolism	Enhanced fermentable substrate availability	FFAR2/3 → AMPK activation → SREBP-1c suppression	Inhibition of hepatic lipid synthesis and promotion of fatty acid oxidation	Contributes to reduced hepatic lipid accumulation	Ríos-Covián et al., 2016; Makki et al., 2018
Microbial taxa	Functional role	Regulation by MACs	Downstream molecules/pathways	Effects on the gut-liver axis	Clinical relevance	–

From the perspective of the gut-liver axis, the regulation of *A. muciniphila* and related taxa by MACs may simultaneously influence intestinal barrier integrity and gut-derived neuroendocrine signaling. If SERT expression or function is reduced, 5-HT reuptake by intestinal epithelial cells may be impaired, leading to elevated extracellular 5-HT levels; a portion of gut-derived 5-HT may then enter the liver via the portal circulation and participate in hepatic lipid metabolism regulation through pathways such as the 5-HT2A receptor (HTR2A) (Wang et al., 2020; Li K. et al., 2024; Belmer et al., 2026). However, causal relationships among MACs, *A. muciniphila*, SERT, 5-HT, and hepatic HTR2A-related pathways remain insufficiently validated and require further investigation. Therefore, this pathway may represent a potential mechanism by which MACs regulate the gut-liver axis, although its definitive role in MAFLD remains to be established.

## 3 Transmucosal transport of gut-derived molecules and portal vein enrichment

Metabolites produced by colonic microbiota exhibit distinct routes of trans-epithelial transport into the systemic circulation. Free luminal molecules must first traverse the intestinal mucosal barrier via selective transport mechanisms and are subsequently drained into the portal vein through the mesenteric venous system (Li et al., 2022; Wang L. J. et al., 2025). Given that portal venous blood is directed first to the liver, hepatocytes are preferentially exposed to gut-absorbed nutrients, microbial metabolites (e.g., SCFAs and bile acids), and gut-derived antigens. This anatomical and hemodynamic configuration enables gut-derived molecules to influence hepatic metabolic and immune microenvironments, thereby providing a mechanistic basis for the initiation and progression of MAFLD as well as for nutritional interventions targeting the gut-liver axis (Zhu et al., 2022; Chen et al., 2023; Ni et al., 2023; Zeng et al., 2023; Dong H. et al., 2025).

### 3.1 Trans-epithelial transport and portal enrichment of SCFAs

Following anaerobic fermentation of MACs by colonic microbiota, SCFAs, primarily acetate, propionate, and butyrate, are generated, typically at an approximate molar ratio of 60:20:20 in most studies. SCFAs can cross the intestinal epithelium via multiple pathways. A small fraction diffuses passively in its undissociated form, whereas the majority is transported through apical membrane transporters, including the proton-coupled monocarboxylate transporter 1 (MCT1, encoded by *SLC16A1*) and the sodium-dependent monocarboxylate transporter 1 (SMCT1, encoded by *SLC5A8*) (Procházková et al., 2023, 2024; Thing et al., 2024).

Once inside colonocytes, butyrate is predominantly utilized in mitochondrial β-oxidation and serves as a primary energy substrate for colonic epithelial cells. Butyrate metabolism increases local oxygen consumption, thereby maintaining a physiologically hypoxic epithelial surface and stabilizing hypoxia-inducible factor 1α (HIF-1α) signaling. This process contributes to the maintenance of tight junction integrity and reduces the risk of portal exposure to harmful luminal molecules (Zhou et al., 2017; Sun et al., 2018; Fang et al., 2019; Yang et al., 2020).

In contrast, acetate and propionate are less extensively metabolized within colonocytes and are more readily transported across the basolateral membrane into the systemic circulation. Consequently, portal venous concentrations of acetate and propionate are typically higher than those in peripheral blood, resulting in preferential hepatic exposure to these gut-derived SCFAs. Once delivered to the liver, acetate and propionate may function as metabolic substrates or signaling molecules and are implicated in the regulation of hepatic lipogenesis, fatty acid oxidation, and associated transcriptional programs (Reuter et al., 2024; Wang L.-Y. et al., 2024).

### 3.2 Microbiota-mediated bile acid transformation and enterohepatic circulation

Beyond SCFAs, bile acids represent key signaling molecules within the gut-liver axis. In addition to their role in lipid digestion and absorption, bile acids regulate lipid metabolism, glucose homeostasis, and inflammatory responses through receptor-mediated signaling pathways. Under physiological conditions, hepatocytes synthesize primary bile acids, including cholic acid (CA) and chenodeoxycholic acid (CDCA), from cholesterol. These bile acids are conjugated with glycine or taurine and secreted into the intestinal lumen via the biliary system. Upon reaching the terminal ileum and colon, conjugated primary bile acids undergo further microbial metabolism and modification.

This process depends on bile salt hydrolase (BSH)-expressing bacteria, which deconjugate primary bile acids by hydrolyzing the amide bond linking bile acids to glycine or taurine, thereby generating unconjugated primary bile acids. These deconjugated bile acids can be further metabolized by bacteria harboring the bile acid-inducible (bai) operon, such as *Clostridium scindens* within Clostridium clusters XI and XIVa. Through 7α-dehydroxylation catalyzed by 7α-dehydroxylase, these microbes convert primary bile acids into hydrophobic secondary bile acids, including deoxycholic acid (DCA) and lithocholic acid (LCA) (Shi et al., 2023; Wise and Cummings, 2023).

In MAFLD-associated gut dysbiosis, alterations in the distal colonic environment may promote the expansion of 7α-dehydroxylating bacteria, thereby increasing the production of hydrophobic secondary bile acids such as DCA. Accumulation of DCA has been associated with hepatic lipid deposition, impaired glucose metabolism, and inflammatory responses (Devlin and Fischbach, 2015; Funabashi et al., 2020; Wang et al., 2023; Wahlström et al., 2024). These secondary bile acids can be reabsorbed in the intestine and recirculated to the liver via the enterohepatic circulation and portal vein. Tracer studies further indicate that bile acids exhibit distinct absorption, distribution, and recycling efficiencies within the enterohepatic system (Voronova et al., 2020; Fuchs and Trauner, 2022; Sudo et al., 2024; Zheng et al., 2024).

Recent evidence suggests that gut microbiota not only mediate classical bile acid transformations but also generate novel derivatives, including amino acid-conjugated bile acids (AA-BAs). Among these, tryptophan–cholic acid conjugates (Trp-CA) have been identified as microbiota-derived metabolic signals that regulate host glucose homeostasis via the orphan receptor MRGPRE. This finding indicates that gut microbiota may establish non-classical signaling networks distinct from the FXR/TGR5 axis through interactions between bile acid and amino acid metabolism. Given that tryptophan metabolism is closely linked to gut-derived 5-HT production, whether AA-BAs participate in coordinated regulation of neuroendocrine signaling and hepatic lipid metabolism remains to be further investigated (Lin et al., 2025).

Upon hepatic entry, bile acids act as endogenous ligands for farnesoid X receptor (FXR) and the G protein-coupled bile acid receptor TGR5, thereby regulating lipid metabolism and inflammatory responses. SCFAs derived from MAC fermentation can lower colonic luminal pH and may suppress 7α-dehydroxylation activity, thereby influencing the ratio of primary to secondary bile acids (Devkota et al., 2012; Trompette et al., 2014; Delannoy-Bruno et al., 2021; Arifuzzaman et al., 2022). Alterations in bile acid composition subsequently engage the FXR–SHP axis to mediate negative feedback regulation of bile acid synthesis and may modulate lipogenic transcription factors such as SREBP-1c (Tokuhara, 2021; Tang Y. et al., 2022; Do et al., 2023; Shi et al., 2026). In addition, certain bile acids may regulate inflammatory responses in Kupffer cells and hepatic sinusoidal endothelial cells via the TGR5–cAMP pathway (Jiao et al., 2021). Collectively, modulation of gut microbiota and bile acid metabolism represents a key mechanism by which MACs influence the gut-liver axis and MAFLD progression.

### 3.3 Intestinal mucosal barrier and portal metabolic endotoxemia

Accumulating evidence indicates that gut microbial dysbiosis contributes to chronic low-grade inflammation and metabolic inflammation, thereby linking obesity, insulin resistance, and MAFLD (Tilg et al., 2020). During MAFLD development and its progression to non-alcoholic steatohepatitis (NASH), impairment of the intestinal mucosal barrier is considered a critical event (Wu and Li, 2020; Wu et al., 2023; Asghari et al., 2025). When dietary intake of fermentable complex carbohydrates or MACs is insufficient, certain gut bacteria may shift toward utilizing mucin glycoproteins from the host mucus layer as an alternative substrate, a process known as mucin-foraging. Excessive mucin degradation can reduce mucus layer thickness and compromise barrier function (Jia et al., 2025; Liu et al., 2026). Concurrently, low-grade inflammation and microbial dysbiosis may alter the expression and distribution of tight junction proteins, such as ZO-1 and Occludin, thereby increasing intestinal epithelial permeability.

Following barrier disruption, LPS and other PAMPs can more readily translocate across the epithelium into the portal circulation, thereby increasing the risk of metabolic endotoxemia (Tang et al., 2024; Wang Z. et al., 2025). In addition to free metabolites and endotoxins, gut microbiota-derived extracellular vesicles (GMEVs) have recently emerged as potential long-range signaling carriers in gut–liver communication. GMEVs can encapsulate LPS, bacterial proteins, nucleic acids, and metabolite-associated molecules, and possess the ability to traverse epithelial barriers. Under conditions of impaired intestinal integrity, a subset of GMEVs may enter the liver via the portal vein and be taken up by Kupffer cells and other hepatic immune cells, thereby activating inflammatory signaling pathways. Emerging studies suggest that GMEVs may contribute to enhanced hepatic inflammation and lipid metabolic dysregulation in metabolic dysfunction-associated steatohepatitis (MASH), potentially serving as a mechanistic link between gut microbial dysbiosis and hepatic immune activation (Kwak et al., 2026). These gut-derived inflammatory mediators can further stimulate Kupffer cells and other hepatic immune populations to produce TNF-α, IL-1β, and IL-6, thereby exacerbating hepatic inflammation, insulin resistance, and lipid metabolic disorders (Xu et al., 2023).

MAC fermentation increases SCFA production, particularly butyrate, thereby supporting intestinal barrier integrity (Ni et al., 2023; Zhu et al., 2023; Miao et al., 2024). The protective effects of butyrate on the intestinal barrier are mediated through three major mechanisms. First, butyrate serves as a primary energy source for colonocytes, enhancing mitochondrial β-oxidation and oxygen consumption, thereby maintaining physiological epithelial hypoxia and limiting expansion of facultative anaerobes. Second, butyrate acts as a histone deacetylase (HDAC) inhibitor, regulating tight junction gene expression and maintaining levels of barrier proteins such as ZO-1 and Occludin. Third, SCFAs signal through free fatty acid receptors on epithelial and goblet cells to promote mucin 2 (MUC2) expression, thereby enhancing mucus layer integrity (Bao et al., 2020; Zhao et al., 2020; Peng et al., 2024).

Therefore, the supportive effects of MACs on intestinal barrier function are mediated through increased SCFA production, maintenance of tight junction architecture, and promotion of mucus layer renewal. Improvement of barrier integrity reduces portal exposure to LPS and other inflammatory mediators, thereby attenuating hepatic inflammatory activation and potentially slowing MAFLD progression. Gut microbiota-derived signaling systems and their portal-mediated regulatory roles in the MAFLD gut-liver axis are summarized in Table 3.

**TABLE 3:** Gut microbiota–derived signaling systems and their portal vein–mediated regulatory roles in the metabolic dysfunction-associated fatty liver disease (MAFLD) gut-liver axis.

Signal category	Representative molecules/systems	Major origin and trans-epithelial transport features	Key receptors/pathways	Main effects on MAFLD	Relevant characteristics	References
SCFAs	Acetate, propionate, butyrate	Produced via MAC fermentation by colonic microbiota; transported across epithelium mainly via MCT1 and SMCT1; acetate and propionate preferentially enter portal circulation	FFAR2, FFAR3, AMPK, HDAC, HIF-1α	Promote fatty acid oxidation, suppress lipogenesis, improve insulin sensitivity, enhance intestinal barrier function	Canonical portal-enriched metabolic signaling system	Bao et al., 2020; Zhu et al., 2023; Liao et al., 2026
Bile acid signaling system	CA, CDCA, DCA, LCA	Primary bile acids undergo microbial deconjugation and 7α-dehydroxylation to form secondary bile acids; recirculated via enterohepatic circulation into portal vein	FXR, TGR5, SHP	Regulation of bile acid homeostasis, lipid metabolism, and inflammatory responses	Core gut-liver enterohepatic signaling axis	Bourgin et al., 2021; Arifuzzaman et al., 2022
Amino acid–conjugated bile acids (AA-BAs)	Tryptophan–cholic acid conjugates (Trp-CA), etc.	Microbial co-metabolism of bile acids and amino acids; reabsorbed into systemic circulation via intestinal absorption	MRGPRE	Regulation of glucose homeostasis and potential effects on hepatic lipid metabolism	Emerging microbiota-derived bile acid–like signaling molecules	Samuel et al., 2008; Lin et al., 2025
Tryptophan metabolites	Indole, IAA, IPA, etc.	Produced by gut microbial tryptophan metabolism; absorbed through intestinal epithelium into portal circulation	AhR, PXR	Maintenance of intestinal barrier integrity, suppression of inflammation, improvement of metabolic homeostasis	Key link between microbial metabolism and host immune regulation	Bhattarai et al., 2018; Hu M. et al., 2025
Gut-derived 5-HT	5-HT	Synthesized by enterochromaffin cells under microbial and metabolite regulation; partially enters liver via portal vein	SERT–HTR2A–PPARγ axis	Regulation of lipogenesis, energy metabolism, and inflammatory responses	Neuroendocrine component of the gut-liver axis	Yano et al., 2015; Choi et al., 2018
Microbial extracellular vesicles (GMEVs)	Bacterial EVs	Nano-sized vesicles released by gut microbiota; capable of crossing epithelial barriers and entering portal circulation	TLR4/NF-κB, MAPK pathways	Activation of hepatic immune cells, promoting inflammation and metabolic dysregulation	Emerging long-range microbial communication system	Kwak et al., 2026
Endotoxin-related signals	LPS and other PAMPs	Translocation across impaired intestinal barrier into portal circulation	TLR4/MyD88/NF-κB	Activation of Kupffer cells, induction of metabolic inflammation and insulin resistance	Central driver of metabolic endotoxemia	Cani et al., 2007; Wang Z. et al., 2025
Protein fermentation metabolites	Ammonia, phenol, p-cresol, hydrogen sulfide, etc.	Generated from microbial proteolytic fermentation of proteins and amino acids; partially absorbed into portal vein	Oxidative stress and inflammatory pathways	Impair intestinal barrier, increase hepatic metabolic burden, promote inflammation	Elevated under MAC deficiency	Yuan et al., 2019; Lee et al., 2020; Chen et al., 2021; Li et al., 2021; Tan et al., 2023
Intestinal barrier regulatory system	MUC2, ZO-1, occludin, claudins	Regulated by SCFAs and microbial metabolites controlling mucus layer and tight junction integrity	HIF-1α, HDAC, FFAR2/3	Reduces translocation of LPS and other gut-derived molecules	Key determinant of portal exposure level and barrier integrity	Bao et al., 2020; Zhao et al., 2020; Peng et al., 2024

## 4 Regulation of hepatic metabolism and inflammatory responses by gut-derived signals

Gut-derived molecules entering the liver via the portal vein, including SCFAs, bile acids, and certain neuroendocrine-related mediators, can achieve relatively high local concentrations within the hepatic microenvironment and elicit cell type–specific metabolic and immunological effects. Given that portal blood flow is first directed to the liver, these molecules are preferentially exposed to periportal regions, hepatic sinusoids, and distinct hepatic cell populations (Zhao et al., 2022; Cha et al., 2025; Zhang et al., 2025). Among these, hepatocytes are primarily responsible for lipid synthesis, fatty acid oxidation, and bile acid metabolism, whereas Kupffer cells represent a central component of innate immune responses in the liver. Accordingly, upon entering the hepatic compartment, gut-derived signals may regulate lipid accumulation and inflammatory processes in MAFLD through receptor engagement, intracellular signaling cascades, and transcriptional modulation.

### 4.1 Hepatocytes: regulation of lipid metabolism pathways by SCFAs

Hepatocytes are the central functional units of hepatic lipid metabolism and the primary cellular site of lipid droplet accumulation and lipotoxic injury in MAFLD. SCFAs produced by colonic microbial fermentation, particularly acetate and propionate, are absorbed through the intestinal mucosa and transported via the portal vein, resulting in preferential hepatic exposure to these gut-derived metabolites (Janssen et al., 2017; Song and Zhang, 2022; Ni et al., 2023; Zhu et al., 2023). In addition to serving as energy substrates, SCFAs function as signaling molecules that may regulate hepatic and intrahepatic cellular metabolic pathways through G protein-coupled receptors, including FFAR2/GPR43, FFAR3/GPR41, and GPR109A (Lu et al., 2016; Overby and Ferguson, 2021; Wang N. et al., 2024).

In several experimental models, SCFAs have been shown to interact with these receptors and activate energy-sensing pathways such as AMP-activated protein kinase (AMPK). Activation of AMPK subsequently suppresses the activity of lipogenic transcription factors, including sterol regulatory element-binding protein 1c (SREBP-1c) and carbohydrate response element-binding protein (ChREBP). This cascade further downregulates the expression of key lipogenic enzymes, such as fatty acid synthase (FAS) and acetyl-CoA carboxylase (ACC), thereby reducing de novo lipogenesis (DNL) (Dai et al., 2021; Hu et al., 2023).

Although acetate has generally been associated with improved metabolic homeostasis through FFAR2/AMPK signaling, it can also serve as a substrate for acetyl-CoA synthesis and *de novo* lipogenesis under conditions of excessive energy availability (Fang et al., 2022; Li W. et al., 2024; Sankarganesh et al., 2025). Therefore, the metabolic effects of acetate are context-dependent and influenced by substrate concentration, dietary background, and host metabolic status. In contrast, butyrate and propionate exhibit more consistent hepatoprotective effects across experimental models.

**FIGURE 2:**
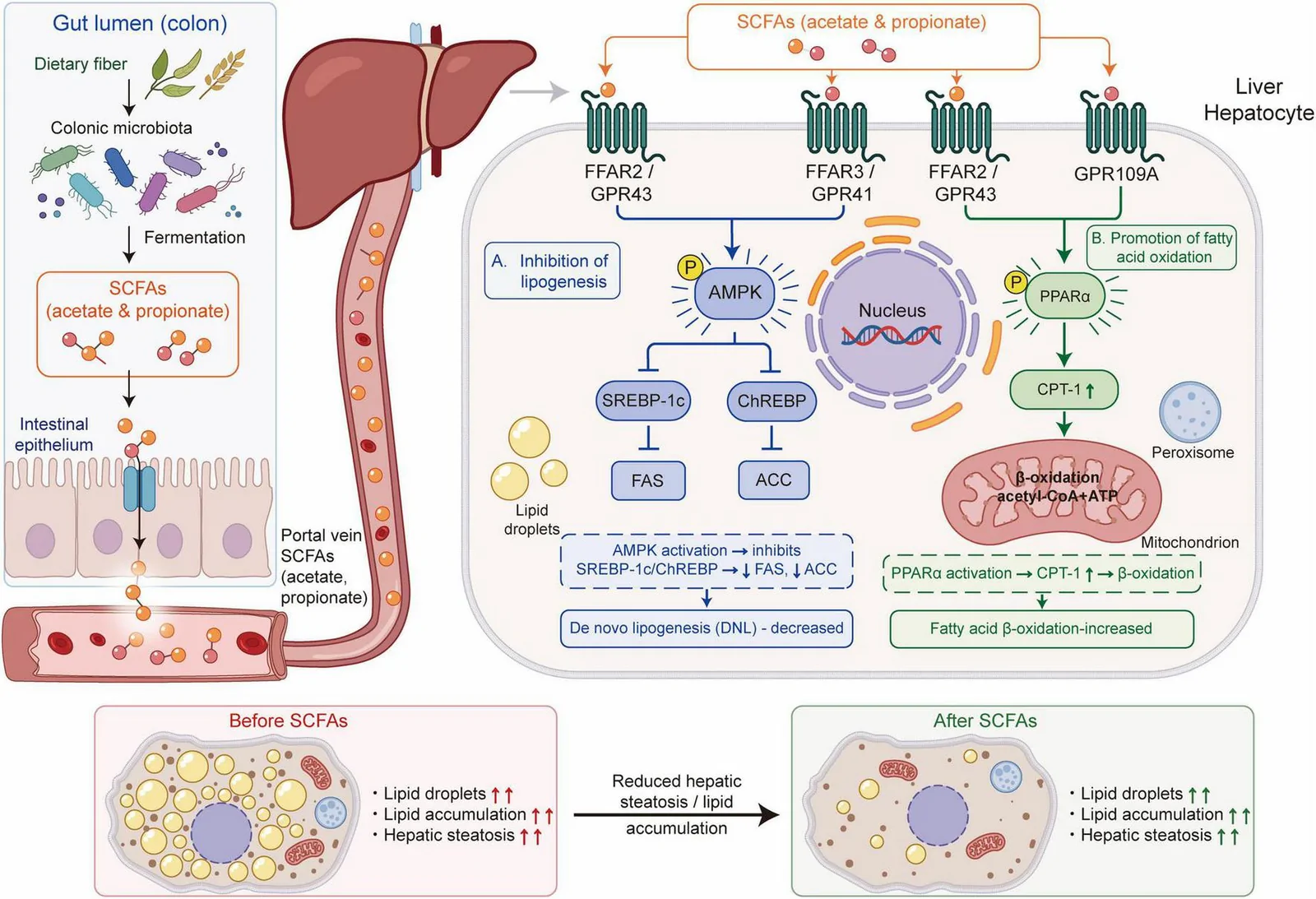
Regulation of hepatic lipid metabolism in metabolic dysfunction-associated fatty liver disease (MAFLD) by short-chain fatty acids (SCFAs) via AMP-activated protein kinase (AMPK) and peroxisome proliferator-activated receptor α (PPARα) signaling pathways.

In addition, SCFAs may modulate peroxisome proliferator-activated receptor α (PPARα)-related signaling pathways (Zhu et al., 2022; Dong Z. et al., 2025). Activation of PPARα upregulates fatty acid oxidation genes, including carnitine palmitoyltransferase 1A (CPT1A), thereby promoting the transport of long-chain fatty acids into mitochondria and facilitating β-oxidation (Kei et al., 2023, 2024; Liu et al., 2024). Collectively, under specific metabolic contexts, SCFAs may attenuate hepatocellular lipid accumulation by suppressing lipogenesis and enhancing fatty acid oxidation (Figure 2). However, the magnitude of these effects and the specificity of underlying targets vary among different SCFA species, and their *in vivo* relevance in human MAFLD requires further investigation.

### 4.2 Kupffer cells: effects of gut-derived signals on hepatic sinusoidal inflammation

Kupffer cells (KCs) are the major resident macrophages of the liver and play a central role in maintaining hepatic sinusoidal immune homeostasis and orchestrating inflammatory responses. During the progression of MAFLD, gut-derived LPS and other PAMPs, together with intrahepatic lipotoxic signals, may synergistically act on KCs to promote their inflammatory activation. Activated KCs subsequently release pro-inflammatory cytokines, including TNF-α, IL-6, and IL-1β, and induce or generate reactive oxygen species and other oxidative stress–related mediators, thereby exacerbating hepatic insulin resistance, dysregulated lipid metabolism, and hepatic inflammatory injury (Li et al., 2019; Xu et al., 2021, 2023; Wu et al., 2022; Tian et al., 2024).

When dietary MAC intake and microbial fermentation are sufficient, butyrate and other SCFAs produced by the gut microbiota may preserve intestinal barrier integrity, reduce systemic exposure to gut-derived inflammatory mediators, and modulate the hepatic sinusoidal immune microenvironment through receptor-mediated and epigenetic mechanisms (Zhao et al., 2020; Tang C. et al., 2022; Werlinger et al., 2022). Butyrate is not only a key energy substrate for colonic epithelial cells but also functions as a signaling molecule regulating macrophage activity. Previous studies have demonstrated that butyrate modulates macrophage inflammatory responses via receptors such as GPR109A (Lee et al., 2020, 2021; Nguyen et al., 2022; Cao et al., 2023), and additionally suppresses NF-κB–dependent inflammatory signaling through inhibition of HDACs (Sun et al., 2018; Zhao et al., 2021; Ren et al., 2022; Zhou et al., 2025a).

**FIGURE 3:**
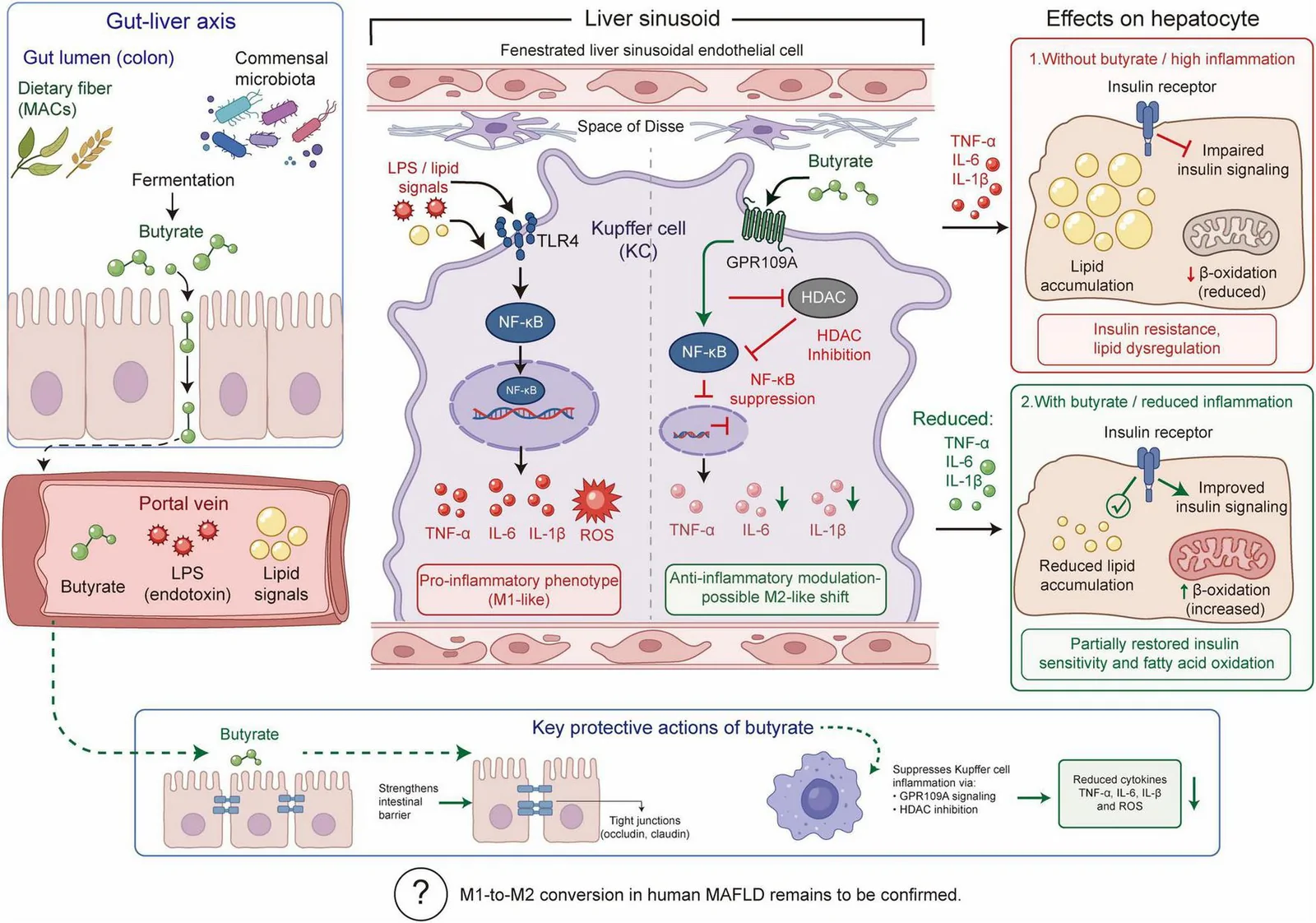
Butyrate regulation of Kupffer cell–mediated inflammation in metabolic dysfunction-associated fatty liver disease (MAFLD) via GPR109A activation and histone deacetylase (HDAC) inhibition.

Mechanistically, these processes may manifest as attenuation of pro-inflammatory activation in KCs, reduced secretion of TNF-α and IL-6, and overall suppression of inflammatory signaling within the hepatic sinusoidal microenvironment. As inflammatory signaling diminishes, hepatocellular insulin signaling and fatty acid oxidation capacity may be partially restored. Therefore, MACs may, at least in part, attenuate MAFLD-associated inflammation through increased SCFA production, acting concurrently at the levels of intestinal barrier maintenance and hepatic immune modulation (Figure 3). Nevertheless, whether KCs undergo stable inflammatory phenotype switching in this context, and the magnitude of such effects in human MAFLD, remain to be further substantiated with direct evidence.

### 4.3 Regulation of lipid metabolism and inflammation via bile acid receptors FXR/TGR5

Bile acids modified by the gut microbiota not only participate in lipid digestion and absorption but also function as signaling molecules involved in the regulation of hepatic metabolism and immune responses. After undergoing enterohepatic circulation and returning to the liver, distinct bile acid species can activate nuclear receptor FXR and G protein-coupled bile acid receptor TGR5, thereby contributing to the regulation of MAFLD-associated lipid accumulation and inflammatory processes.

Within hepatocytes, a subset of bile acids with FXR agonistic activity can activate FXR signaling. FXR activation subsequently induces the expression of small heterodimer partner (SHP) (Bourgin et al., 2021; Dong Z. et al., 2025; Zhao et al., 2025). SHP, on the one hand, suppresses cholesterol 7α-hydroxylase (CYP7A1) expression, thereby mediating negative feedback regulation of bile acid synthesis; on the other hand, it may downregulate lipogenic transcriptional programs, including SREBP-1c, thereby reducing *de novo* lipogenesis (Ridlon and Gaskins, 2024; Lucas et al., 2026). Accordingly, the FXR–SHP axis represents a central pathway linking bile acid signaling to hepatic bile acid homeostasis and lipid metabolism.

G protein-coupled bile acid receptor is expressed in multiple non-parenchymal liver cell types, including Kupffer cells and liver sinusoidal endothelial cells. Bile acids with TGR5 agonistic activity may activate the adenylate cyclase–cAMP–protein kinase A (PKA) signaling cascade, thereby suppressing NF-κB–mediated inflammatory signaling and reducing the production of pro-inflammatory cytokines such as TNF-α and IL-1β (Dhakal and Dey, 2022; Kastl et al., 2022; Reuter et al., 2024). This process may attenuate the pro-inflammatory activation state of hepatic sinusoidal immune cells and alleviate local inflammatory responses (Fleishman and Kumar, 2024).

**FIGURE 4:**
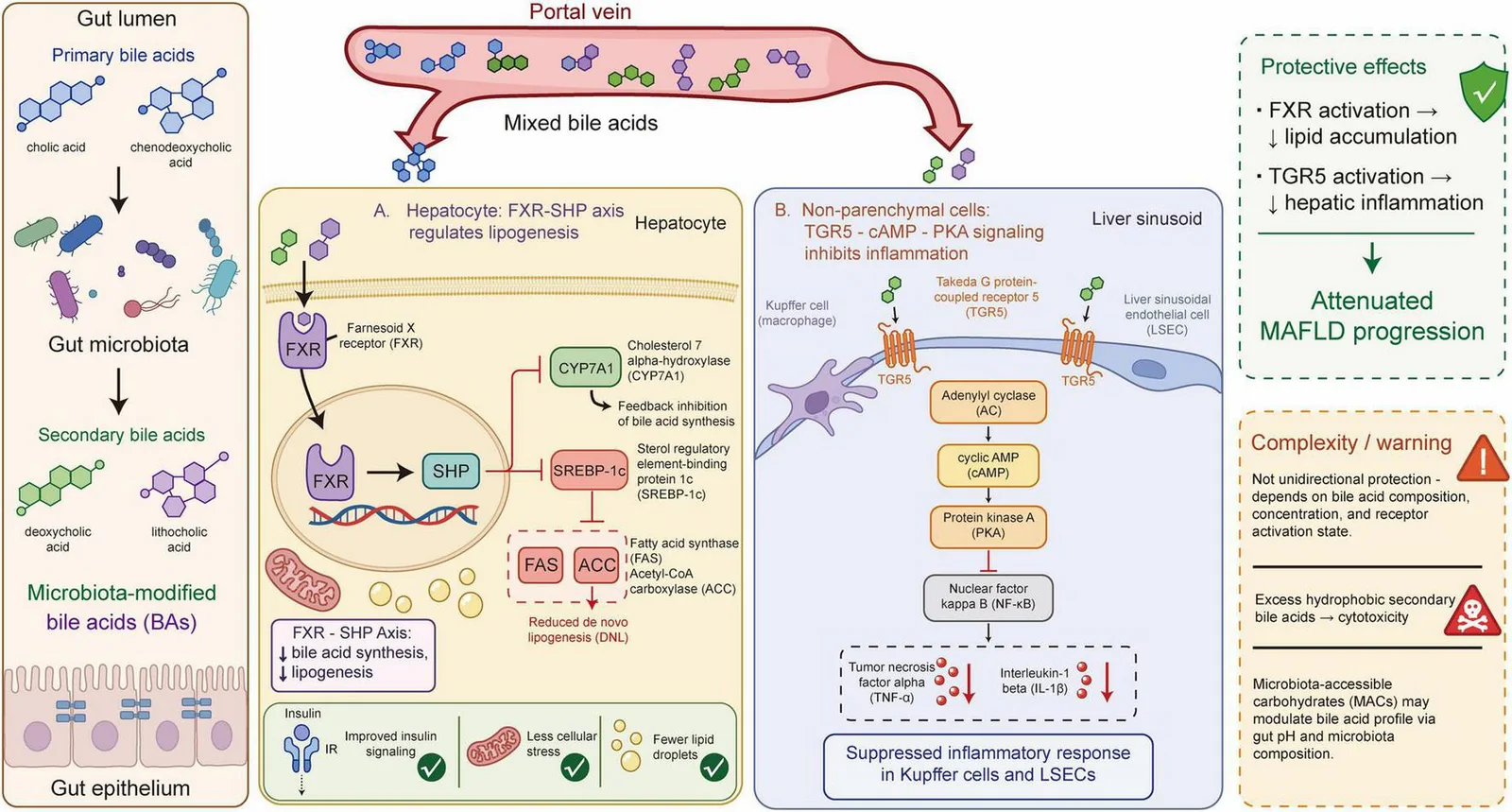
Microbiota-modified bile acids regulate hepatic lipid metabolism and inflammation in metabolic dysfunction-associated fatty liver disease (MAFLD) via farnesoid X receptor (FXR) and TGR5 signaling pathways.

Collectively, microbiota-driven alterations in bile acid composition may influence hepatic lipid metabolism and sinusoidal immune responses through FXR- and TGR5-dependent pathways, respectively. If MACs reshape bile acid metabolism by modulating gut microbial composition, luminal pH, and the activity of bile acid-transforming bacterial taxa, they may thereby further influence MAFLD progression. However, bile acid signaling does not exert uniformly protective effects. Excess accumulation of certain hydrophobic secondary bile acids may induce cytotoxicity, and the overall outcome depends on bile acid composition, concentration, and the activation status of FXR/TGR5-mediated signaling pathways (Figure 4).

### 4.4 Gut-derived 5-HT signaling and hepatic lipid metabolism

In recent years, the mechanisms by which SCFAs and bile acids regulate hepatic metabolism via the gut-liver axis have been extensively characterized. In contrast, the role of gut-derived 5-HT in MAC-mediated regulation of MAFLD remains incompletely understood. Given the critical functions of 5-HT in glucose and lipid metabolism, immune regulation, and energy homeostasis, this section proposes a theoretical framework—based on established gut–liver signaling paradigms of SCFAs and bile acids—in which gut-derived 5-HT participates in intestinal–hepatic communication and modulates MAFLD progression, while integrating current evidence to explore its potential mechanisms.

5-hydroxytryptamine is primarily synthesized and secreted by enterochromaffin cells in the gut, and its local concentration is tightly regulated by the expression and function of the SERT (encoded by SLC6A4). SERT mediates the reuptake of extracellular 5-HT and plays a critical role in maintaining intestinal 5-HT homeostasis (Li G. et al., 2024). Evidence suggests that in MAFLD or metabolic dysregulation, the abundance of *A. muciniphila* in the gut may be reduced (Juárez-Fernández et al., 2021; Rao et al., 2021; Nian et al., 2023). The outer membrane protein Amuc_1100 of Akkermansia muciniphila can interact with Toll-like receptor 2 (TLR2) on intestinal epithelial cells, thereby contributing to mucosal barrier maintenance and local immune regulation (Wang et al., 2021). Under certain experimental conditions, changes in *A. muciniphila* abundance may be associated with alterations in SERT expression. If SERT function is impaired, extracellular intestinal 5-HT levels may increase, potentially leading to enhanced portal vein exposure and subsequent hepatic delivery of gut-derived 5-HT (Yang et al., 2023; Li K. et al., 2024).

Once 5-HT enters the liver, it may act on hepatocytes via the HTR2A receptor (a Gq/11-coupled receptor), inducing intracellular Ca^2 +^ signaling and modulating MAPK pathway activity. Previous studies have demonstrated that gut-derived 5-HT may regulate PPARγ signaling through HTR2A, thereby contributing to the regulation of hepatocellular lipid metabolism (Choi et al., 2018). PPARγ is involved in the regulation of lipid uptake, storage, and metabolic homeostasis, and its activity may influence both fatty acid oxidation and lipid synthesis. In addition, 5-HT signaling may also affect inflammatory and fibrogenic responses in hepatic non-parenchymal cells, such as Kupffer cells and hepatic stellate cells, thereby contributing to MAFLD progression (Yan et al., 2023; Belmer et al., 2026).

**FIGURE 5:**
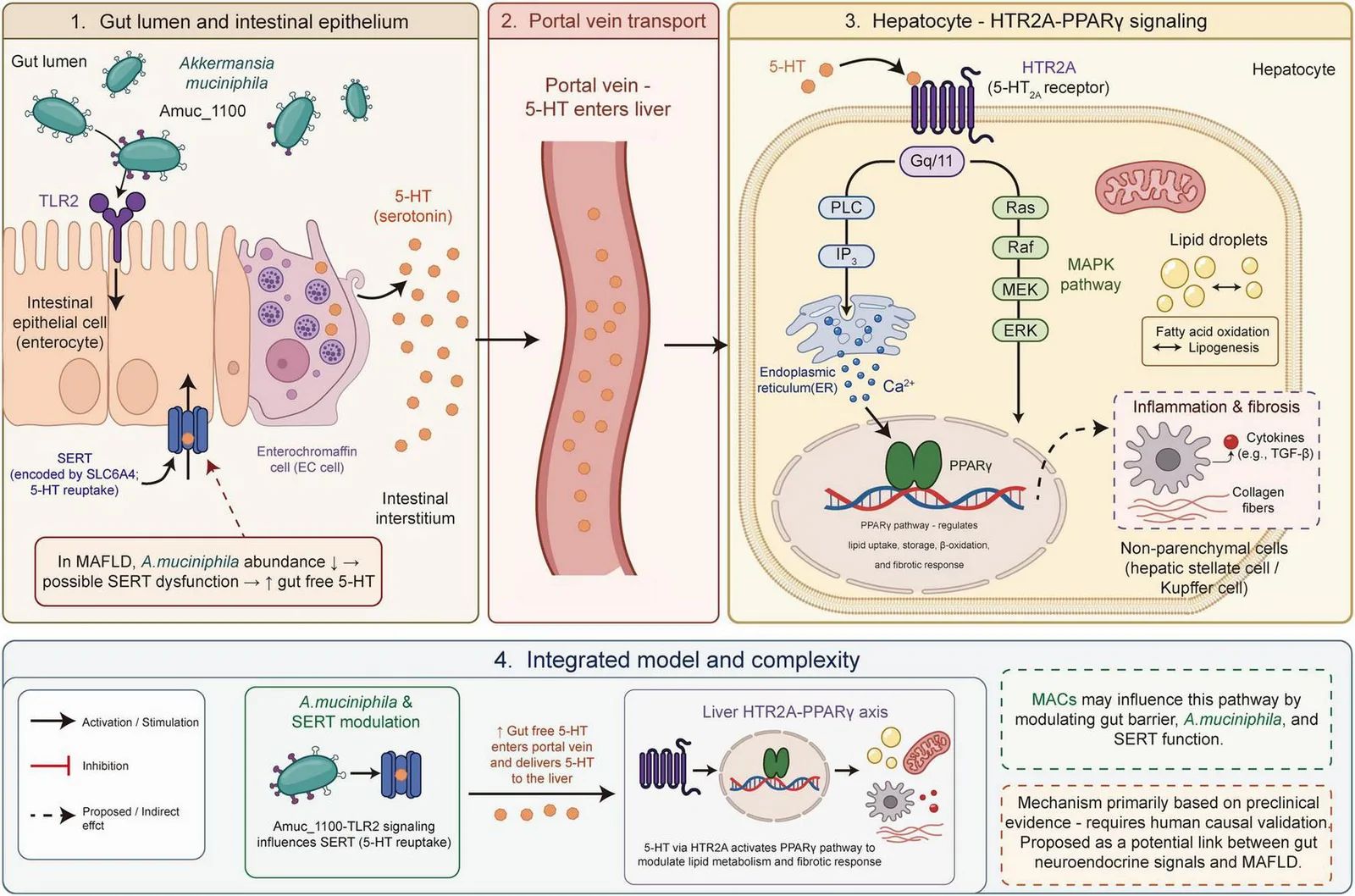
Putative mechanisms by which gut-derived 5-hydroxytryptamine (5-HT) regulates hepatic lipid metabolism via the HTR2A-PPARγ signaling axis in metabolic dysfunction-associated fatty liver disease (MAFLD).

Accordingly, MACs may indirectly influence hepatic HTR2A–PPARγ signaling by modulating *A. muciniphila* abundance, intestinal mucosal barrier homeostasis, and SERT-mediated 5-HT metabolism (Figure 5). This framework provides a conceptual basis for linking gut microbiota, neuroendocrine signaling, and hepatic lipid metabolism, and suggests a novel direction for future investigation. At present, direct causal evidence linking all components of the proposed MACs–*Akkermansia*–SERT–5-HT–HTR2A axis in a single MAFLD model is still lacking. Existing evidence supports several individual links, whereas the complete signaling cascade remains hypothetical and requires future validation.

## 5 Limitations and future perspectives

Although nutritional interventions targeting the gut-liver axis represent a promising strategy for MAFLD management, accumulating evidence suggests that microbiota-accessible carbohydrates (MACs) may beneficially modulate hepatic metabolism through microbial remodeling, metabolite production, and intestinal barrier regulation. Representative MACs and current evidence supporting their potential benefits in MAFLD are summarized in Table 4. However, current evidence remains largely derived from animal studies, in vitro models, and mechanistic investigations. The major limitations of MAC-based interventions and corresponding future research directions are summarized in Table 5.

**TABLE 4:** Representative Microbiota-accessible carbohydrates (MACs) and current evidence for their potential benefits in metabolic dysfunction-associated fatty liver disease (MAFLD).

MAC type	Representative sources	Evidence related to MAFLD	Evidence level	References
Inulin-type fructans	Chicory root, onion, garlic, asparagus	Promote beneficial bacteria and SCFA production; improve hepatic steatosis, insulin resistance, inflammation, and intestinal barrier dysfunction in animal models. Human trials support improvements in metabolic parameters, although direct liver outcomes remain limited.	Strong	Bao et al., 2020; Yang et al., 2023; Amadieu et al., 2025
Resistant starch (RS)	Legumes, green banana, high-amylose maize starch	Enhance butyrate-producing bacteria and improve glucose/lipid metabolism in NAFLD models. Human studies demonstrate beneficial effects on insulin sensitivity, lipid profiles, and microbiota composition.	Strong	Dhakal and Dey, 2022; Zhu et al., 2022; Ni et al., 2023
β-glucans	Oats, barley, mushrooms	Modulate gut microbiota, bile acid metabolism, and inflammatory responses. Clinical evidence supports improvements in cholesterol metabolism and metabolic risk factors associated with MASLD.	Moderate–strong	Kei et al., 2023, 2024; Liu et al., 2024
Pectin	Citrus fruits, apples	Increase SCFA production and improve intestinal barrier function; protective effects against hepatic steatosis have been reported in experimental models. Human MASLD-specific evidence remains limited.	Moderate	Chen X. et al., 2022; Hu et al., 2023; Liu et al., 2026
Arabinoxylans	Wheat bran and cereals	Promote beneficial microbial fermentation and SCFA production; preliminary evidence suggests improved metabolic abnormalities. Human liver-specific evidence is insufficient.	Emerging	Deehan et al., 2017; Gowd et al., 2019
GOS/FOS	Functional foods and vegetables	Mainly studied for microbiota modulation and SCFA production; potential metabolic benefits have been reported, but direct evidence for MAFLD improvement is limited.	Limited	Deehan et al., 2017; Gowd et al., 2019

**TABLE 5:** Limitations of **Microbiota-accessible carbohydrates (**MACs)-based interventions and future research directions.

Limitation/issue	Identified cause in the text	Proposed improvements	Potential technologies/ approaches	References
Species differences between animal models and humans	Differences in cecal size, transit time, and fiber-degrading microbiota composition	Use human-relevant colonic fermentation systems or organ-on-chip platforms	SHIME *in vitro* colon model; human gut-liver organ-on-a-chip systems	Procházková et al., 2023
Difficulty in portal vein sampling	Ethical constraints; peripheral blood does not fully reflect portal exposure	Combine animal models with hepatic metabolomics, single-cell and spatial transcriptomics	scRNA-seq; spatial transcriptomics	Zhu et al., 2022
Inter-individual microbiome variability	Distinct enterotypes (*Prevotella* vs. *Bacteroides* dominance)	Stratified intervention based on CAZyme profiles and metabolomic features	Stable isotope tracing of MAC metabolic fate	Martinez-Medina et al., 2021
Safety concerns of probiotic modulation	Excessive mucin degradation by *Akkermansia muciniphila* may impair barrier integrity	Synbiotic strategies with concurrent substrate supply; dynamic monitoring of barrier and inflammation status	Co-supplementation of fermentable carbohydrates; longitudinal monitoring of mucus layer thickness and inflammatory markers	Cani et al., 2022
Insufficient clinical evidence	Predominance of animal and *in vitro* studies	Human interventional trials with metabolic biomarker tracking	Randomized controlled trials; integrated metabolomics and microbiome profiling	Ni et al., 2023; Miao et al., 2024; Amadieu et al., 2025

### 5.1 Metabolic differences between animal models and humans

Most mechanistic studies investigating the effects of dietary complex carbohydrates (MACs) on hepatic lipid metabolism are based on high-fat diet (HFD)-induced rodent models. However, there are substantial physiological differences between mice and humans, including cecal volume, intestinal transit time, and the composition of fiber-degrading microbial communities (Procházková et al., 2023). Consequently, the fermentation rate, fermentation site, and metabolite profiles of the same MACs may differ significantly between murine and human colonic environments, warranting caution when extrapolating animal findings to human MAFLD.

Future studies should adopt experimental systems that more closely recapitulate human physiology. For instance*, in vitro* gut fermentation platforms such as SHIME, as well as human-derived gut or liver organ-on-a-chip systems, may be integrated to evaluate the fermentation characteristics and metabolic effects of different MACs in human-relevant models, thereby improving the translational validity of preclinical findings.

### 5.2 Limited access to portal vein sampling and insufficient resolution of hepatic local effects

Portal vein exposure of gut-derived metabolites represents a critical step in gut-liver axis communication affecting hepatic metabolism and immune responses. However, direct sampling of portal blood in human studies remains technically and ethically challenging. Most existing studies rely on peripheral blood measurements of metabolites such as SCFAs and bile acids; nevertheless, these circulating levels are substantially influenced by hepatic first-pass metabolism and therefore may not accurately reflect true portal or hepatic sinusoidal exposure (Zhu et al., 2022).

Accordingly, future research should establish standardized portal vein sampling protocols in animal models and integrate multi-omics approaches, including liver tissue metabolomics, single-cell RNA sequencing (scRNA-seq), and spatial transcriptomics. These technologies will enable more precise characterization of how gut-derived metabolites act across distinct hepatic cell populations, facilitating identification of key pathways involved in hepatocellular lipid metabolism and Kupffer cell–mediated inflammatory responses.

### 5.3 Inter-individual variability in gut microbiota influences MAC efficacy

The fermentation and metabolic effects of MACs are highly dependent on the baseline composition of the gut microbiota. Individuals may exhibit distinct enterotypes, such as *Prevotella*-dominant or *Bacteroides*-dominant profiles, which are associated with differences in carbohydrate-active enzyme (CAZyme) repertoires. These variations may influence MAC degradation efficiency, fermentation localization, and SCFA production capacity, thereby contributing to inter-individual variability in dietary intervention outcomes (Martinez-Medina et al., 2021).

Therefore, MAC-based interventions should not rely on uniform or fixed dietary strategies. A more rational approach would integrate baseline microbiome composition, CAZyme profiles, metabolomic signatures, and host metabolic status to enable stratified or personalized interventions. Stable isotope tracing techniques may further be employed to track carbon flux from specific dietary substrates *in vivo*, thereby determining whether particular MACs are efficiently converted into target metabolites and improving the precision and reproducibility of nutritional intervention assessments.

### 5.4 Potential risks of next-generation probiotic interventions

Certain dietary MACs may reshape microbial ecological niches and thereby influence the abundance and activity of *A. muciniphila* and other candidate next-generation probiotics (NGPs). *A. muciniphila* has been associated with intestinal barrier integrity, metabolic homeostasis, and inflammation regulation, and is therefore considered a promising therapeutic candidate. However, its effects are context-dependent and may vary according to host status, substrate availability, and the intestinal ecological environment.

*A. muciniphila* possesses the capacity to utilize host-derived mucin. Under conditions of sufficient fermentable carbohydrate availability, it may contribute to mucus layer renewal; however, under conditions of limited external carbon sources or pre-existing barrier impairment, excessive mucin degradation may further weaken the mucus layer, increase intestinal permeability, and elevate the risk of endotoxin translocation (Cani et al., 2022). Therefore, interventions targeting *A. muciniphila* and other NGPs should be designed in conjunction with substrate availability and should not be implemented independently of dietary context.

Future microbiome-based interventions for MAFLD should prioritize synbiotic strategies that integrate probiotics with fermentable carbohydrate substrates. In addition, dynamic monitoring of intestinal barrier integrity, inflammatory status, and metabolic parameters will be essential for evaluating both efficacy and safety. For next-generation probiotic interventions, outcome measures should extend beyond microbial abundance to include functional activity and host barrier interactions.

## 6 Conclusion

The gut-liver axis represents a critical pathway through which the gut microbiota contributes to the development and progression of MAFLD. Dietary complex carbohydrates, particularly MACs, may participate in the regulation of hepatic lipid metabolism and inflammatory responses by modulating colonic fermentation processes, the profile of gut-derived metabolites, and intestinal mucosal barrier function.

Current evidence suggests that MACs may exert multi-dimensional metabolic regulatory effects on MAFLD by promoting SCFA production, improving bile acid metabolism, reducing the generation of potentially harmful fermentation byproducts, and modulating *A. muciniphila* and 5-HT-related signaling pathways. However, these mechanisms remain to be further validated in human studies. Future research should aim to delineate the effects of distinct MAC types, inter-individual microbiome variability, intervention dosage, and long-term safety profiles, thereby facilitating the translation of MAC-based strategies into clinically applicable nutritional interventions.
